# Rapid, non-contact multifocal visual assessment in multiple sclerosis

**DOI:** 10.1007/s10072-022-06387-z

**Published:** 2022-09-13

**Authors:** Ted Maddess, Joshua P. van Kleef, Emilie M. F. Rohan, Corinne F. Carle, Jonathan Baird-Gunning, Bhim B. Rai, Anne Bruestle, Jo Lane, Christian J. Lueck

**Affiliations:** 1 Eccles Institute of Neuroscience, John Curtin School of Medical Research, Australian National University, Canberra, ACT Australia; 2 Royal North Shore Hospital, Saint Leonards, New South Wales Australia; 3 Department of Immunology and Infectious Disease, John Curtin School of Medical Research, Australian National University, Acton, ACT Australia; 4 National Centre for Epidemiology and Population Health, Australian National University, Canberra, ACT Australia; 5 Department of Neurology, The Canberra Hospital, Canberra, ACT Australia; 6 Australian National University Medical School, Acton, ACT Australia

**Keywords:** Multifocal assessment, Multiple sclerosis, Progressive MS, Optic neuritis, Pupillary response

## Abstract

**Objective:**

Previous work on temporally sparse multifocal methods suggests that the results are correlated with disability and progression in people with multiple sclerosis (PwMS). Here, we assess the diagnostic power of three cortically mediated sparse multifocal pupillographic objective perimetry (mfPOP) methods that quantified response-delay and light-sensitivity at up to 44 regions of both visual fields concurrently.

**Methods:**

One high-spatial-resolution mfPOP method, P129, and two rapid medium-resolution methods, W12 and W20, were tested on 44 PwMS and controls. W12 and W20 took 82 s to test both visual fields concurrently, providing response delay and sensitivity at each field location, while P129 took 7 min. Diagnostic power was assessed using areas under the receiver operating characteristic (AUROC) curves and effect-size (Hedges’ *g*). Linear models examined significance. Concurrent testing of both eyes permitted assessment of between-eye asymmetries.

**Results:**

Per-region response delays and asymmetries achieved AUROCs of 86.6% ± 4.72% (mean ± SE) in relapsing–remitting MS, and 96.5% ± 2.30% in progressive MS. Performance increased with increasing disability scores, with even moderate EDSS 2 to 4.5 PwMS producing AUROCs of 82.1 to 89.8%, Hedge’s *g* values up to 2.06, and *p* = 4.0e − 13. All tests performed well regardless of any history of optic neuritis. W12 and W20 performed as well or better than P129.

**Conclusion:**

Overall, the 82-s tests (W12 and W20) performed better than P129. The results suggest that mfPOP assesses a correlate of disease severity rather than a history of inflammation, and that it may be useful in the clinical management of PwMS.

## Introduction

Measures of regional or global brain atrophy are not well-correlated with progression of disability in multiple sclerosis (MS) [1]. Loss of grey matter is important, but its precise relationship to disability in MS remains unclear, i.e. structure does not correlate precisely with function [2]. It is likely that the incorporation of some form of functional assessment would enhance prognostication and management in MS. Visually evoked potentials (VEPs) represent a form of functional assessment and are still used in diagnosis of multiple sclerosis [3]. In particular, *multifocal* VEPs (mfVEPs) can test multiple parts of the visual fields and, therefore, multiple parts of the visual system concurrently. They have been shown to have diagnostic power related to the overall functional status of people with MS (PwMS), particularly if so-called *temporally sparse* stimuli are employed [4]. Multifocal pupillographic objective perimetry (mfPOP) is a newer, but related, technology. It measures both the amplitude and the latency of the pupillary response to stimulation of many regions of the visual fields of both eyes [5, 6]. Responses from both eyes are obtained concurrently, meaning that asymmetry between anatomically equivalent parts of the two eyes can be assessed, providing sensitive within-individual measures. The extrastriate cortex appears to be involved in generating responses to sparse mfPOP stimuli [7], as supported by neuro-anatomy [8]. Thus, although the mfPOP method was initially designed for use in ophthalmic diseases [9–11], it has also been used to study neurological issues including visual attention [12], migraine [13], epilepsy [14], concussion [15] and multiple sclerosis (MS) [16]. With respect to MS, responses to sparse stimuli may involve a feedback loop in the optic radiations [17], which while representing 1% of the total white matter volume, display 7–10% of the T2 lesion load [18].

Our original cross-sectional study of 85 PwMS indicated that mfPOP results were highly correlated with clinical disease severity [16]. As has also been reported for mfVEPs using sparse stimuli [4], sensitivity and specificity for diagnosing MS did not depend on a history of optic neuritis (ON). These findings suggested that mfVEP and mfPOP might be assessing something correlated with disability rather than simply the history of acute inflammation. A recent 10-year follow-up study using mfPOP indicated that the findings of the original study were predictive of future clinical progression [19]. The mfPOP methods used in the original study have since been superseded by newer versions with much higher signal-to-noise ratios [20]. These methods allow both eyes to be assessed concurrently in less than 7 min and provide greater spatial resolution of the visual fields with test–retest variability that is half that of standard automated perimetry [21].

One consideration is that a more rapid, spatially coarser, assessment of the quadrants of the inner and outer visual fields may be sufficient for use in neurological testing. With this in mind, new mfPOP algorithms have recently been developed with total test durations of just 82 s [22]. This study was set up to compare the diagnostic power of the 7-min and 82-s methods, examining both PwMS and normal-controls. The mfPOP tests were done with an FDA-cleared ObjectiveFIELD Analyzer (OFA).

## Methods

This study was approved by the ACT Health Human Research Ethics Committee (ETH 7.07.667) and Australian National University Human Ethics Committee (2010/194) and conformed to the Declaration of Helsinki. Informed written consent was obtained from all participants.

### Participants

Forty-four people with MS were studied, 31 of whom were female. All PwMS had been diagnosed by a neurologist based upon clinical and laboratory findings. Thirty-one had relapsing–remitting MS (RRMS) and the other 13 had progressive MS. Their ages were 60.7 ± 10.1 years (mean ± SD), range 36 to 78 years. They were part of a cohort of 85 persons who had been studied 10 years earlier [16, 19]. Expanded Disability Status Scale (EDSS) scores were assessed by a neurologist on the same day as mfPOP assessment.

The disease status of PwMS was classified in three different ways: clinical type of MS, EDSS score, and any history of ON. Possible clinical types were relapsing–remitting MS (RRMS), secondary progressive MS (SPMS) or primary progressive MS (PPMS). For some analyses, EDSS scores were divided into 3 groups, each with approximately equal numbers: ≤ 2.5; 2.5 to 4.5; and $\ge$ 4.5.

In addition to mfPOP testing, all participants underwent ocular examination that included best corrected visual acuity (log-MAR), slit-lamp examination, posterior pole and retinal nerve fibre layer optical coherence tomography (OCT, Spectralis, Heidelberg Engineering GmbH, Germany), and Matrix or HFA 24–2 automated perimetry (Carl Zeiss Meditec Inc., Dublin, CA). A prototype of the FDA-cleared ObjectiveFIELD Analyser (OFA) (Konan Medical USA, Irvine, CA) was used for the mfPOP testing.

### mfPOP

**Fig. 1:**
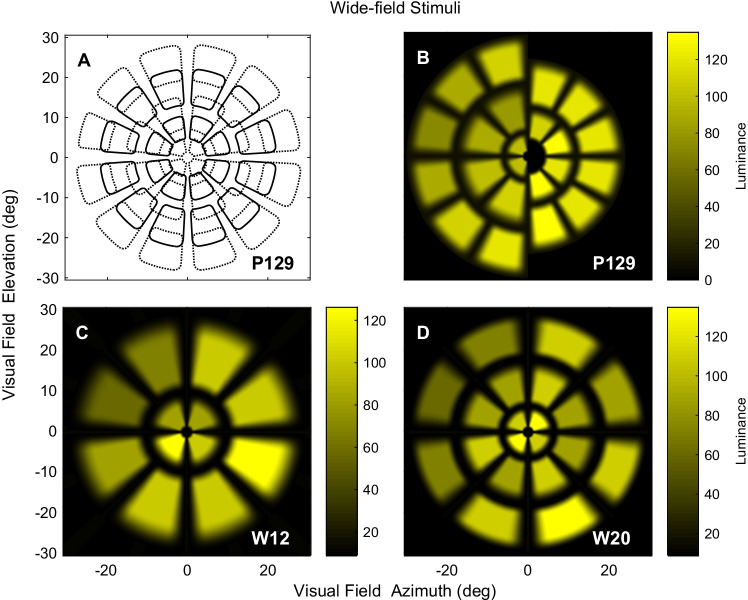
The mfPOP stimulus ensembles used. **A** Contour plot for P129 showing slight overlap of the 44 individual stimuli. **B** Left and right hemifield subsets of the 3- and 2-ring P129 stimuli, respectively, shown without opposite hemifield (right and left) stimuli for clarity. **C** Stimulus arrangement in the W12 test. **D** Stimulus arrangement in the W20 test

The standard mfPOP stimulus is a 44-region/eye, 7-min test referred to as P129. It has previously been reported to have high signal-to-noise ratios [20], reproducibility [21], and diagnostic power [9]. P129 is a wide-field stimulus, extending ± 30° from fixation (Fig. 1A, B). The two new, 82-s stimuli covered the same wide-field area of visual-field but delivered either 12 or 20 stimuli/eye and were therefore labelled W12 and W20 (Fig. 1C, D). All 3 stimulus ensembles respected the horizontal and vertical meridians. Individual stimuli lasted 33 ms and were delivered with a mean inter-stimulus interval of 4 s at any one location, in pseudo-random temporal sequences. In total, 90 stimuli were presented at each location using P129, and the stimuli were delivered so that they never overlapped spatially. W12 and W20 presented 22 stimuli at each location. The per-region responses were thus the mean for the N presentations, 90 or 22.

Figure 1 also illustrates the luminance and colour of the various stimuli. Luminance levels were adjusted to generate similar amplitudes of pupillary response from stimulation of each region of the visual field in normal persons. This luminance normalisation exploited the pupil gain-control system [6] to enhance per-region signal-to-noise ratios (SNR) [23]. Stimulus luminances ranged from 67 to 150 cd/m^2^ in P129, from 49 to 156 cd/m^2^ in W12, and from 53 to 167 cd/m^2^ in W20. The stimulus backgrounds were yellow at 10 cd/m^2^ in all three.

Results of P129 for PwMS were compared to pre-existing normative data obtained from 115 individuals in the same age range (57.8 ± 12.8 year). We also tested 40 age-matched normal controls (60.2 ± 12.5 years) for comparisons using W20 and W12.

### Analysis

Pupillary responses to stimulation at each test region from each participant generated a mean time-to-peak constriction (‘delay’), along with a standardised peak constriction amplitude which was converted to decibels (‘sensitivity’). The response estimation and related multifocal analysis methods have been given previously [24, 25]. Diagnostic power was assessed by comparing PwMS with controls, and by calculating the area under the receiver operating characteristic curve (AUROC). We also measured effect-size using Hedges’ *g* (Hedges’ *g* is Cohen’s *d* corrected for smaller numbers). The accepted cutoffs for effect-sizes for *d* and *g* are: small 0.2, medium 0.5, large 0.8, very large 1.2, and huge 2.0. Similar AUROCs and *g*-values were also derived for different sub-groups of interest.

Our AUROC analysis methods have been published previously [9, 23, 26]. Briefly, the mfPOP analysis extracted the average response to stimulation at each of the 12, 20 or 44 regions per eye for W12, W20 and P129, respectively. The normative values at each region were taken as the median of the normal-controls’ values, regardless of age or sex. We then computed three standard measures for each test:

- Per-region deviations, or *total deviations* (*TDs*), i.e. the differences from the normative values for each individual at each visual-field location.
- *Pattern deviations* (*PDs*). These provide within-individual controlled data by subtracting a response level corresponding to the overall ‘normal’ of that field. To do this, the 86th percentile of the TD data for each visual field was subtracted from TD measurements for all visual-field locations in any given test.
- *Between-eye asymmetry* (*Asymm*). This involves calculating the difference between pairs of test results arising from anatomically equivalent regions of the two eyes, e.g. superior temporal left eye cf. superior temporal right eye. This helps control for inter-individual variability. Although the subject of many scientific studies, *Asymm* has not yet been incorporated into commercial perimeters, probably because existing commercial perimeters test one eye at a time resulting in potential confounding by test order. This confounder does not apply to OFA which tests all regions of both visual fields concurrently.

To investigate how many regions/field might provide good diagnostic power, the measures of interest (TDs, PDs, Asymms) from each visual field stimulus location were sorted from worst (least normal) to best (most normal). Initially, the single worst stimulus location from every participant (both PwMS and controls) was used to generate an AUROC. Thereafter, the means of the worst 2 locations in each field were included in the AUROC analysis. This process was repeated for the means of the worst 3, then 4, and up to 12 regions. Change in AUROC as a function of N-worst locations provides information about the distribution of visual field losses [9, 23, 26]. That is, if a few regions/field are highly diagnostic, then the damage is localised within the field; if data from many regions needs to be combined to generate good diagnostic power, then the damage is diffusely distributed across the field. This study reports the worst 4, and worst 12, regions/field for each of the three measures of interest, looking at the ability of the three methods to classify disease status.

## Results

Of the 44 PwMS, 31 were classified as RRMS, 2 as PPMS and 11 as SPMS. Owing to the small numbers, the two progressive classifications were collapsed for further analysis. The overall average EDSS score was 3.81 ± 2.00 (mean ± SD) and the scores for the RRMS and progressive MS groups were 2.8 ± 1.32 (mean ± SD) and 6.2 ± 1.03, respectively. Twenty-three out of 31 (74%) RRMS participants and 7/13 (54%) participants with progressive disease had a history of ON. Table 1 shows the AUROC values (% ± SE) and Hedges’ *g* looking at the 4- and 12-worst region delays of each individual’s visual fields, looking at PDs and Asymms, for each of the three mfPOP tests, broken down by diagnostic classification, i.e. RRMS (Table 1 (A)) and progressive MS (Table 1 (B)). Analysis using TDs did not perform as well as PD or Asymm values, and so, these results are not reported. AUROCs ranged from 73 to 87% for the RRMS group and 86 to 97% for the progressive group. All *g* values for RRMS were > 0.8 (‘large’) while those of the progressive group were mostly in the in the ‘huge’ range.

**Table 1:** Area under receiver operator characteristic (AUROC) curves for relapsing–remitting and progressive MS groups, looking at the 4- and 12-worst per-region delays of individuals’ visual fields and comparing P129, W12 and W20 mfPOP algorithms

		AUROC (% ± SE)		Hedges’ *g*	
		*N* = 4	*N* = 12	*N* = 4	*N* = 12
A. Relapsing–remitting					
P129	Pattern dev	72.6 ± 5.22	75.6 ± 4.74	1.10	1.16
	Asymm	75.7 ± 5.32	78.6 ± 4.95	1.28	1.46
W12	Pattern dev	77.3 ± 5.52	77.5 ± 5.47	0.93	0.95
	Asymm	83.0 ± 4.97	82.2 ± 4.81	1.05	0.99
W20	Pattern dev	79.8 ± 5.51	78.0 ± 5.57	1.11	1.05
	Asymm	86.6 ± 4.72	83.9 ± 4.73	1.35	1.26
B. Progressive					
P129	Pattern dev	94.7 ± 3.47	93.4 ± 2.86	2.86	2.69
	Asymm	96.2 ± 1.76	97.0 ± 1.75	3.09	3.32
W12	Pattern dev	93.4 ± 3.52	91.6 ± 6.57	1.93	1.81
	Asymm	96.5 ± 2.30	94.5 ± 3.79	2.07	2.02
W20	Pattern dev	85.9 ± 6.55	86.6 ± 6.42	1.85	1.87
	Asymm	93.0 ± 3.42	94.3 ± 3.47	2.54	2.49

In all analyses using delays, Asymm performed better than PD. W20 performed better than W12 or P129 in the RRMS group, but it performed less well in those with progressive MS. Combining RRMS and progressive groups and comparing them to controls for the 4 worst regions, Asymm data for W12 yielded an AUROC of 87.0% ± 3.85% and a Hedges’ *g* of 1.11 (‘large’). A similar analysis for W20 yielded an AUROC of 87.3% ± 3.79% and a Hedges’ *g* of 1.45 (‘very large’). For P129 the values were 81.7% ± 4.10% and 1.52 (‘very large’). Thus, the pooled values are intermediate between those in Table 1 (A and B). Analysis of PD revealed similar results (not shown).

Table 2 shows an analysis for three approximately equal-sized EDSS severity groups, examining the Asymm data for delays for the three mfPOP methods. The AUROC increased with increasing disability for all three tests. All Hedges’ *g* values were ≥ 1.12 (‘very large’). For W12, the moderately severe EDSS 2.5 to 4.5 group produced an %AUROC of 89.8 ± 4.74%.

**Table 2:** Area under receiver operator characteristic (AUROC) curves and Hedges’ *g* values for the 4- and 12-worst per-region delay asymmetries of individuals’ visual fields as a function of increasing EDSS severity, comparing P129, W12 and W20 mfPOP algorithms

	EDSS	AUROC (% ± SE)		Hedges’ *g*	
		*N* = 4	*N* = 12	*N* = 4	*N* = 12
P129	≤ 2.5	73.9 ± 7.00	77.7 ± 5.80	1.12	1.25
	2.5 to 4.5	80.5 ± 6.62	82.9 ± 6.48	1.73	2.06
	≥ 4.5	90.3 ± 6.19	91.1 ± 6.13	2.74	2.93
W12	≤ 2.5	75.0 ± 8.33	76.4 ± 7.53	1.12	1.12
	2.5 to 4.5	89.8 ± 4.74	88.4 ± 4.58	1.70	1.62
	≥ 4.5	95.4 ± 2.62	93.3 ± 3.77	1.91	1.88
W20	≤ 2.5	81.1 ± 6.69	81.5 ± 6.00	1.32	1.26
	2.5 to 4.5	86.5 ± 6.26	84.6 ± 6.03	1.85	1.70
	≥ 4.5	94.1 ± 3.12	94.4 ± 3.16	2.37	2.35

Table 3 illustrates the impact of the very large effect-sizes on the significance of the differences between EDSS levels. The table shows the results of linear models comparing the means of the 4-worst delay Asymm data for control subjects and the 3 EDSS severity groups of Table 2. Here, the estimates for W20 were most significant, achieving *p* = 4.00E − 13 for the EDSS 2.5 to 4.5 group. Given the means of the 4-worst regions and the cyclopean data, there was 1 data point per subject.

**Table 3:** Summary of linear models for W12 (upper) and W20 (lower) rapid stimulus methods. The models fitted the mean of the 4 biggest delay asymmetries (Asymm) to factors for normal controls and the 3 grades of EDDS severity from Table 3. Thus, the estimates for the 3 EDDS groups are differences compared to control subjects, and the significance of those differences is indicated by the *t*- and *p* values. Age and sex are also fitted but are non-significant

Parameter	Estimate (ms)	SE (ms)	*t*-stat	*p* value
W12				
Controls	− 18.5	2.25	− 8.21	-
EDSS ≤ 2.5	− 7.7	3.32	− 2.32	0.022
EDSS 2.5 to 4.5	− 16.8	3.25	− 5.17	6.64E − 07
EDSS ≥ 4.5	− 30.8	3.27	− 9.42	3.76E − 17
Sex	1.9	2.49	0.78	0.438
Age	− 1.7	1.02	− 1.68	0.095
W20				
Controls	− 35.4	2.88	− 12.30	-
EDSS ≤ 2.5	− 15.4	4.23	− 3.64	0.0004
EDSS 2.5 to 4.5	− 32.7	4.15	− 7.88	4.00E − 13
EDSS ≥ 4.5	− 39.8	4.18	− 9.53	1.91E − 17
Sex	4.2	3.18	1.33	0.186
Age	− 0.8	1.30	− 0.60	0.551

Table 4 shows the results of categorisation by a history of ON or not. The Hedges’ *g* values for delays were generally larger in participants who had not had a history of ON. This was also true for the AUROC results for *N* = 12, but not *N* = 4.

**Table 4:** Area under receiver operator characteristic (AUROC) curves and Hedges’ *g* values for the 4- and 12-worst delay asymmetries of individuals’ visual fields as a function of history of optic neuritis (Hx ON or no Hx ON), comparing P129, W12 and W20 mfPOP algorithms

		AUROC (% ± SE)		Hedges’ *g*	
		*N* = 4	*N* = 12	*N* = 4	*N* = 12
P129	No Hx ON	81.1 ± 6.85	86.2 ± 5.33	1.99	2.02
	Hx ON	82.0 ± 4.89	83.0 ± 4.79	1.63	1.73
W12	No Hx ON	86.2 ± 5.77	86.9 ± 5.24	1.50	1.45
	Hx ON	87.4 ± 4.62	86.0 ± 4.46	1.29	1.29
W20	No Hx ON	84.5 ± 6.44	87.2 ± 5.45	1.69	1.75
	Hx ON	88.7 ± 4.06	86.8 ± 4.36	1.63	1.50

Looking at sensitivity as opposed to the previous analyses, which all utilised delay, analysis of TDs yielded AUROCs of > 85% for PwMS with progressive disease. The result was consistent with the fact that these individuals were likely to have higher EDSS scores and so have experienced more neural degeneration. For individuals with less marked disability, however, sensitivity provided little useful diagnostic power compared to delay.

## Discussion

This study investigated the performance of three different versions of mfPOP (W12, W20, and P129) in a population of people with multiple sclerosis (PwMS) using three different per-region measures (total deviations, pattern deviations, and between-eye asymmetries). The results showed that analysis of delay (time-to-peak) of pupil response was superior to analysis of sensitivity (amplitude). Analysis of interocular asymmetry (Asymm) performed better than pattern deviations (PD), and both performed much better than total deviations (TD). The 82-s mfPOP versions yielded AUROCs of 83.0 to 86.6% in RRMS participants, and 93.0% to 96.5% in those with progressive disease (with Hedges’ *g* > 2.0, ‘huge’). AUROCs were greater in participants with higher EDSS scores, but were 89.8 ± 4.74 for W12 for those PwMS in the moderate disease severity group who had scores of 2.5 to 4.5. This resulted in very high significance levels (Table 3). Overall, the performances of the W12 and W20 (82-s) versions of mfPOP were roughly equivalent, and both outperformed the 7-min P129 version. While AUROCs were unaffected by the presence or absence of a history of ON, Hedges’ *g* results were considerably larger in those participants without a history of ON, especially when the mean of the 12 most delayed visual field regions was considered.

These findings are consistent with previous reports that mfVEPs [4] and mfPOP [16] are able to discriminate well between PwMS and controls, particularly PwMS who have higher EDSS scores. Importantly, the two testing algorithms that lasted only 82 s performed as well, or better, than the original algorithm (P129) which lasted 7 min [16]. This strongly suggests that mfPOP has the potential to be useful in the clinical management of PwMS as a quick, reliable, inexpensive marker of MS which can assist in diagnosis and in monitoring of treatment efficacy. In addition, mfPOP has recently been shown to have predictive power in relation to disease progression at 10 years [19].

Our earlier studies indicated that mfVEPs produced higher sensitivity and specificity for MS when the stimuli used were made progressively more temporally sparse [4], and that diagnostic power was also just as high for eyes with and without a history of ON. These sparse stimuli produced VEPs that were 15 times larger than conventional methods [4], and sparse stimuli in mfPOP have been shown to perform similarly [16]. We have previously suggested that the increased response-gain seen with sparse stimuli arises from a form of cortico-thalamic feedback [17]. There are over three times as many efferent axons travelling from the cortex to the lateral geniculate nucleus (LGN) as there are afferent axons travelling from LGN to cortex [27]. Thus, sparse stimuli might be testing four times as many axons as those tested by conventional VEP stimuli. This would enhance diagnostic sensitivity by increasing the chance of detecting small lesions. Of note, while the optic radiations make up about 1% of the total white matter volume of the brain, T2 lesions in the optic radiations in PwMS represent 7–10% of the total T2 lesion load [18]. In parallel mfVEP/mfPOP studies we have shown that mfPOP responses include strong extrastriate cortical input [7], which is supported by many neuro-anatomical studies [8].

Classical VEPs have only been shown to demonstrate abnormalities in eyes with a history of ON [4]. Similar to our previous mfPOP studies [16] and other pupillographic studies of MS [28], this study found that the presence or absence of a history of ON did not significantly affect the ability of mfPOP to distinguish individuals with MS from controls. If anything, the test performed better in individuals without a history of ON. mfPOP testing has also been shown elsewhere to be predictive of MS disease progression [19]. These two findings strongly suggest that mfPOP is detecting something related to overall disability rather than simple inflammation-related damage to the optic nerve. The explanation for this might lie in the fact that mfPOP appears to be assessing the extensive connections between extrastriate cortex and LGN, as discussed above. This is clearly worthy of further study.

The two within-individual control measures: within-eye pattern deviation, and between-eye asymmetry performed best, especially the latter, suggesting that asymmetry might be the most clinically useful measure. The ability to test between-eye asymmetry by studying both eyes concurrently gives mfPOP a potential advantage over other methods of visual assessment in PwMS. Both shorter tests (W12 and W20) performed as well, or better than the longer test (P129) on almost all measures. W12 and W20 each had some advantages. W12 generated somewhat higher AUROC scores in the progressive group, but W20 performed better in the relapsing–remitting group. There was little difference in the performance of W12 and W20 when comparing different EDSS groups or whether or not there was a history of ON. Overall, the findings of this study, combined with the independent finding that mfPOP results are predictive of development of progressive disease 10 years later [19], suggest that W12 or W20 might play a useful role in the clinical monitoring of MS over time.

The number of individuals in this study was small, and it is important to validate the findings in a larger, prospective group. However, the consistently high AUROC values, along with the ‘very large’ to ‘huge’ Hedges’ *g* scores, strongly suggest the results are robust. Much current MS research is directed towards improving biomarkers that can assist in diagnosis, monitoring and prognosis of the disease. Multiple clinical, image based, and blood- or CSF-based biomarkers already exist [29], but they are either complex and costly with limited availability (such as MRI scanning), or are invasive and potentially non-specific, at least when looked at in isolation (such as neurofilament light chain [30]). mfPOP clearly shows promise as a clinically meaningful biomarker since it represents a quick, non-invasive, easily available, and inexpensive adjunct to MRI scanning. It can be used for more frequent clinical assessment than MRI, and studies are currently ongoing to determine how it and other biomarkers are best employed to optimise diagnosis, management and prognosis of PwMS at an individual level.

## Conclusions

The newer mfPOP algorithms allow objective, non-invasive assessment of PwMS in 82 s. The diagnostic power is high, meaning that mfPOP has potential as a clinically useful marker of disease in MS. Similar performance for eyes with and without a history of optic neuritis strongly suggests the test is assessing something related to disease severity/progression rather than just a history of inflammation. This is consistent with the finding that an older version of mfPOP has demonstrated ability to predict disease progression over 10 years [19].

## Data Availability

The data that supports the findings of this study are available upon request from Prof Maddess.
